# LOCATION MODERATES THE EFFECT OF COGNITIVE TRAINING ON CRASH RISK OVER 20 YEARS: AN ANALYSIS OF THE ACTIVE STUDY

**DOI:** 10.1093/geroni/igad104.2179

**Published:** 2023-12-21

**Authors:** Katie Wheeler, Tyler Bell, Brooke Carroll, Caitlin Pope, Karlene Ball, Olivio Clay, Despina Stavrinos, Sheila Black

**Affiliations:** University of Alabama at Birmingham, Birmingham, Alabama, United States; University of California, San Diego, San Diego, California, United States; University of Alabama at Birmingham, Birmingham, Alabama, United States; University of Kentucky, Lexington, Kentucky, United States; University of Alabama at Birmingham, Birmingham, Alabama, United States; University of Alabama at Birmingham, Birmingham, Alabama, United States; University of Alabama at Birmingham, Birmingham, Alabama, United States; The University of Alabama, Tuscaloosa, Alabama, United States

## Abstract

Driving is a critical everyday function that helps older adults remain independent. The Advanced Cognitive Training in Vital Elderly (ACTIVE) study has shown cognitive training improves driving safety outcomes for older adults. Few studies have examined the longitudinal effects of cognitive training on crash risk beyond 5 years. To address this empirical gap, we aimed to determine the effects of cognitive training on crash risk over 20 years, and how urbanicity moderates these relationships. Using participants from the no-contact control and intervention (speed of processing, memory, and reasoning training) groups in the ACTIVE study (n=2170) and state-recorded crash reports, cox proportional hazards models were used to examine the effect of three cognitive training interventions as predictors of time to first at-fault crash over 20 years. Participants (mean age 73, 73% White, 23% Male) reported driving 90.6 miles/week on average and 12.6% experienced an at-fault crash. Overall, speed of processing training was the only intervention related to lower crash risk, but this varied by urbanicity. Namely, speed of processing training was related to lower crash risk for individuals living in rural environments (HR=.16, 95% CI [.04-.73], p=.018) but not for individuals in urban environments (HR=.89, 95% CI [.54-1.50], p=.679). Findings highlight the safety benefits of speed of processing training for older drivers living in rural environments where more fatal crashes occur. However, interventions are still needed to improve safety in urban environments where more crashes occur generally. Consideration of location in future interventions may help improve safe driving for older adults.

